# Detection of Vero Cells Infected with Herpes Simplex Types 1 and 2 and Varicella Zoster Viruses Using Raman Spectroscopy and Advanced Statistical Methods

**DOI:** 10.1371/journal.pone.0153599

**Published:** 2016-04-14

**Authors:** Mahmoud Huleihel, Elad Shufan, Leila Zeiri, Ahmad Salman

**Affiliations:** 1 Department of Microbiology, Immunology and Genetics, Faculty of Health Sciences, Ben-Gurion University of the Negev, Beer-Sheva, Israel; 2 Department of Physics, SCE- Shamoon College of Engineering, Beer-Sheva, Israel; 3 Department of Chemistry, Ben-Gurion University of the Negev, Beer-Sheva, Israel; Cincinnati Childrens Hospital Medical Center, UNITED STATES

## Abstract

Of the eight members of the herpes family of viruses, HSV1, HSV2, and varicella zoster are the most common and are mainly involved in cutaneous disorders. These viruses usually are not life-threatening, but in some cases they might cause serious infections to the eyes and the brain that can lead to blindness and possibly death. An effective drug (acyclovir and its derivatives) is available against these viruses. Therefore, early detection and identification of these viral infections is highly important for an effective treatment. Raman spectroscopy, which has been widely used in the past years in medicine and biology, was used as a powerful spectroscopic tool for the detection and identification of these viral infections in cell culture, due to its sensitivity, rapidity and reliability. Our results showed that it was possible to differentiate, with a 97% identification success rate, the uninfected Vero cells that served as a control, from the Vero cells that were infected with HSV-1, HSV-2, and VZV. For that, linear discriminant analysis (LDA) was performed on the Raman spectra after principal component analysis (PCA) with a leave one out (LOO) approach. Raman spectroscopy in tandem with PCA and LDA enable to differentiate among the different herpes viral infections of Vero cells in time span of few minutes with high accuracy rate. Understanding cell molecular changes due to herpes viral infections using Raman spectroscopy may help in early detection and effective treatment.

## Introduction

One of the major causes of serious and life-threatening diseases in humans and animals are viruses. HSV-1, HSV-2 and VZV, which belong to the herpes family of viruses, are responsible for different human infections. They are mainly involved in painful and uncomfortable cutaneous infections; and in some cases can cause serious disorders such as blindness in the case of eye infection, and even death in the case of brain infections. That is in addition to their involvement in serious genital infections [1]. Clinically, there is a high degree of similarity between the symptoms of infections from these viruses to those of bacterial or fungal infections. Therefore, it is very important to identify the cause of the infection rapidly and reliably, thereby enabling the physician to target the infection with the most appropriate treatment to avoid medical complications and side effects.

The routinely used detection assays of herpes viruses are cell culture, immunoassays [2] and molecular techniques which are usually time consuming and expensive. Apart from these conventional methods of herpes infection diagnosis [2, 3] there is a need to develop new approaches that are simple, objective, and noninvasive. Among the optical methods available, Raman spectroscopy has shown encouraging trends in the field of medicine. Raman spectroscopy is a noninvasive tool for studying biological systems that is well known for its simplicity and rapidity [4–7].

Analyzing biomolecules using Raman spectroscopy has become a promising tool for their detection and identification. Furthermore, there is no need for special sample preparation such as drying, labeling, or different fixation, which enables measuring biological samples with minimal manipulations and damage. The Raman technique has already been used for detection and identification of different kinds of cancers like melanoma [8], breast cancer [9, 10], squamous cell carcinoma [11], human coronary atherosclerosis [12], individual neoplastic and normal hematopoietic cells [13], uterine cervical cancer [14, 15], basal cell carcinoma [16], and skin cancer [17]. That is in addition to the identification of biochemical changes due to cell proliferation cultures [18, 19] and discrimination between normal and malignant cells in culture [20–25].

Raman shifts are characteristic to the vibrational molecular modes [26, 27] of the examined sample. The measured spectrum is considered as a ‘biochemical fingerprint’ because it contains bands that represent all molecules within the tested region of the sample [28]. The high spatial resolution of Raman spectroscopy (~ 1 μm) provides qualitative and quantitative information on the biochemical composition and structure of cells and tissues [29–32].

Various biomolecular components of the cell give a characteristic spectrum, which is rich in structural and functional aspects [22, 33]. The biochemical fingerprint of cells, tissues, and fluids that have been altered in a diseased state can be detected using Raman spectroscopy [34–39]. In our previous work [40] we used Raman spectroscopy followed by advanced statistical methods to successfully differentiate, with sensitivity approaching 100%, between a control group of Vero cells and another group of Vero cells that had been infected with HSV-1.

The main purpose of this work is to use Raman spectroscopy as an objective method for characterization and identification of Vero cells infected with herpes simplex viruses HSV-1, HSV-2, and VZV in cell culture. Cell cultures are considered as an advantageous and more convenient model for basic research [41, 42] when compared to “real” tissues, due to their homogeneity and the ability to control important culture parameters such as growth and malignant transformation rate. Cell cultures are used as a complementary method for studying the vibrational modes of normal and infected cells. The obtained Raman data will be studied focusing on the characteristic spectroscopic differences between the various herpes viral infections. These differences will then be used to identify and characterize the infected cells with each of the tested viruses.

## Materials and Methods

### 2.1 Cells and viruses

African green monkey kidney (Vero) cells were obtained from the American Type Culture Collection (ATCC), Rockville, MD, USA. The cells were grown in RPMI medium containing 10% fetal calf serum (FCS), 1% glutamine, 50 U per ml penicillin, and 50 ug per ml streptomycin. The ambient temperature was 37°C in humidified air containing 5% CO_2_. Herpes viruses HSV-1, HSV-2, and VZV, were propagated to > 10^9^ plaque forming units (PFU) per ml in Vero cells. Concentrations were estimated by plaque assay [43].

### 2.2 Cell infection and estimation of viral infection

Vero cells were plated at 0.20 million per well in 24 well culture plates in RPMI, with 10% FCS and antibiotics. After overnight incubation, the medium was removed and the wells were divided into four groups. Three of the four groups were infected, in RPMI containing 2% FCS for 2 hours at 37°C, by one of the three different viruses (HSV-1, HSV-2 and VZV), at a multiplicity of infection (m.o.i.) of 1. The fourth group was devoted as controls. The unadsorbed virus particles were removed and fresh medium containing 2% FCS was added, the monolayers thereafter incubated at 37°C. At 24 hours post infection the infected cells were examined by the following methods:

1. Raman spectroscopy
2. Morphological examination for the appearance of the cytopathic effect (CPE), which is defined as areas of complete destruction of cells or of morphologically modified cells are done by light inverted microscope. The percentage of damaged cells in the inspection field expresses the amount of the CPE
3. MTT test.

In order to evaluate the number of living cells we used the MTT test. This test is an assay that examines the metabolic activity of the cells by measuring the reduction of 3-(4,5-dimethylthiazol-2-yl)-2,5-diphenol tetrazolium bromide (MTT) in the tested cells [44].

### 2.3 Sample Preparation

The cells were picked up from the tissue culture plates by treatment with trypsin (0.25%) for 2–3 minutes. The cells were centrifuged at 1000 rpm for five minutes. The pellet was washed twice with a physiologic solution (saline) and re-suspended in 100 μl of saline. The cells were counted by a hematocytometer, pelleted again by centrifugation, and re-suspended in an appropriate volume of saline to give a concentration of 40–50 cells per μl. Three microliters of the investigated cells were mounted on quartz slides and were measured in vitro using the Raman microscope after air drying for 15 minutes.

### 2.4 Raman measurements

A Jobin-Yvon (JY) LabRam HR 800 micro-Raman system with a liquid nitrogen cooled detector was used to carry out the Raman measurements in the 200–4000 cm^-1^ wavenumber region. Each measurement took 2 minutes to be performed. An argon laser (514 nm) was used for excitation, giving 3mW power on the sample. The specifications of the Raman system were determined to achieve about 4cm^-1^ spectral resolution. A 100 μm microscope with confocal hole and a microscope objective of x100 were used. A 600 grids per mm grating was set when performing the measurements. The measurements were performed over several weeks and the samples were prepared from different batches of cell cultures.

### 2.5 Spectral analysis

We tested 22 uninfected Vero cells, 21 cells infected with HSV-1 (Vero-HSV-1), 23 cells infected with HSV-2 (Vero-HSV-2), and 21 cells infected with VZV (Vero-VZV). All the spectra were cut in the 600–1800 cm^-1^ region and were baseline corrected using concave rubberband correction after normalization with the vector normalization method, and then offset corrected using commercial OPUS 7 software.

#### 2.5.1 Smoothing

Savitzky-Golay algorithm was used with 13 points in order to smooth the spectra. A small number of points were used to keep all the features of the Raman spectrum, without distortion of narrow bands.

#### 2.5.2 Baseline correction

Concave rubberband correction algorithm was used for baseline correction. Using this method, the spectrum was divided into 64 ranges that have the same size. The minimum intensities in each range were calculated. A polynomial function was fitted using the calculated minima and was subtracted from the spectrum to achieve the baseline corrected spectrum.

#### 2.5.3 Normalization

By using vector normalization, the average intensity at all wavenumbers of the spectrum is calculated and subtracted from the spectrum. The obtained spectrum is normalized to 1 by calculating the sum of the squares of all y-values, and dividing the spectrum by the square root of this sum. After performing vector normalization, some of the intensities of the spectrum are negative. Thus the all the vector normalized spectra were offset corrected by shifting the minimal intensities to zero.

### 2.6 Statistical analysis

Our objective was to analyze the Raman spectra of the measured cells to determine its type. The cell type belongs to one of the four categories—the control Vero, and three types of infected cells—Vero-HSV-1, Vero-HSV-2, and Vero-VZV. Each Raman spectrum includes *D* points (several hundreds)—Raman shift intensity as a function of wavenumber. In this study we acquired N = 217 measurements from the four categories. Each measurement is represented as a point in a D-dimensional space. PCA was used to reduce the number of dimensions, followed by LDA, which was used to detect characteristic features of each category [45–48].

#### 2.6.1 LOO algorithm

The separation validation was carried out using the LOO algorithm. It is a common method of cross-validation that is used in case of a small-sized population. It has been extensively explored in machine learning. Using this method, the training set contains (N-1) measurements and one measurement is left out for validation. The procedure is repeated N times, and for each repetition a different measured spectrum is left out. All the measurements are used both for training and for validation with no randomness role. Using the LOO algorithm it is possible to calculate the average number of successes.

## Results

**Fig 1:**
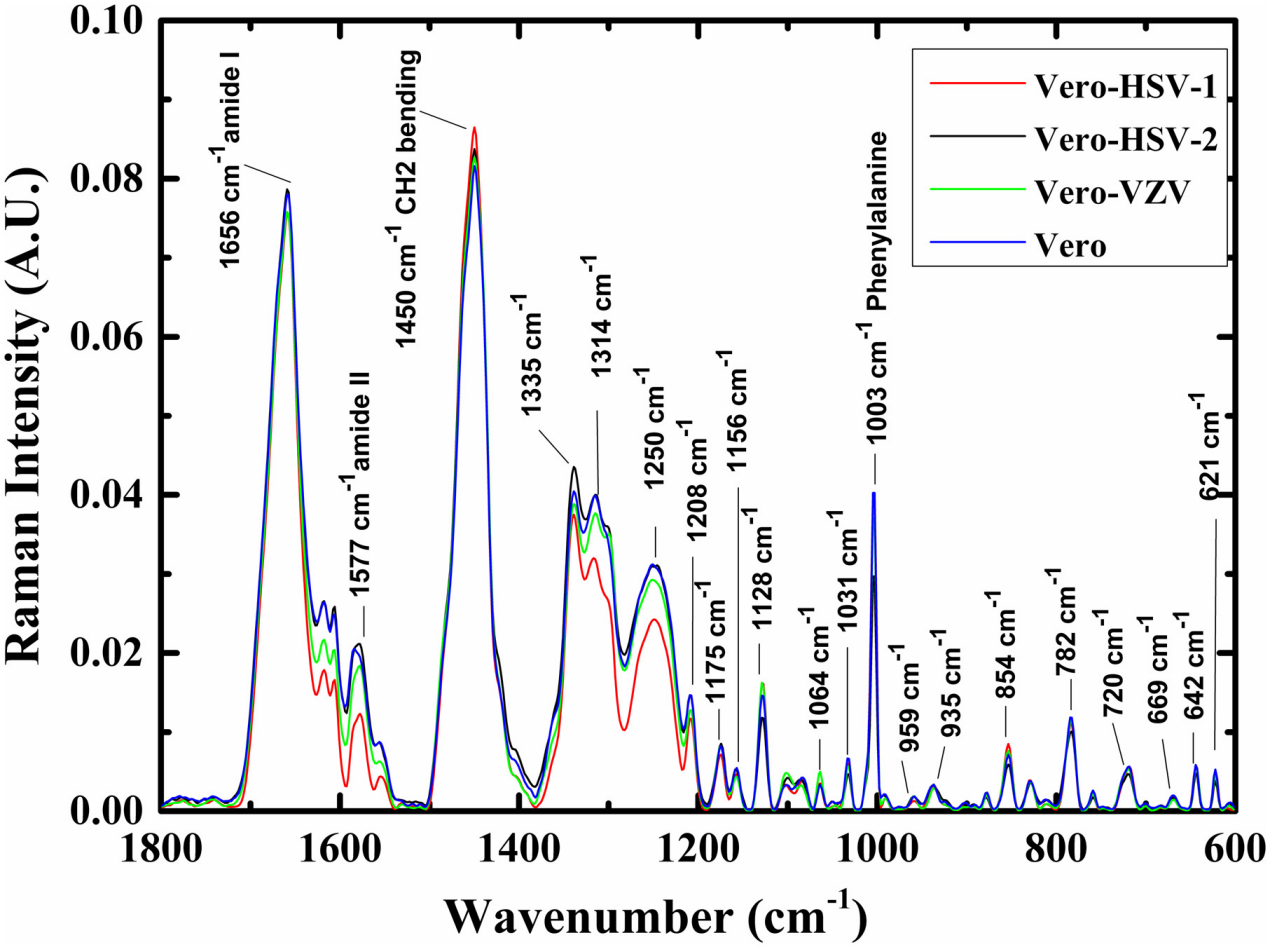
Average spectra of Raman shift bands. Four average spectra of Vero cells, HSV-1 infected Vero cells, HSV-2 infected Vero cells, and VZV infected Vero cells in the 600–1800 cm^-1^ range. All the spectra were manipulated and normalized using OPUS software. The major Raman shifts are labeled in the figure.

Typical Raman spectra of control Vero cells, HSV-1, HSV-2, and VZV infected Vero cells, are presented in Fig 1 in the 600–1800 cm^-1^ wavenumber range. Each spectrum is an average of about 40 measurements taken from cells.

Proteins, lipids, nucleic acids, and carbohydrates are the main molecules that are included in all biological samples. The absorptions of these molecules' functional groups compose the Raman spectra of the four biological samples investigated in this study. Proteins are the main contributor to wavenumber region 1488–1726 cm^-1^, due to amide I and II bands [49–52], with centroids at 1656 cm^-1^ and 1577 cm^-1^, respectively. The peak centered at 1640 is attributed to amide I [53], while the peak centered at 1627 cm^-1^ is attributed to amide C = O, stretching vibration of β-form [54]. Phenylalanine, tyrosine aromatic portions, and to other proteins C = O stretching vibration [13, 55] attribute mainly to the shift bands at 1606 and 1618 cm^-1^. Phenylalanine and hydroxyproline are the main contributors to the Raman band, due to the stretching vibration C—C, centered at 1580 cm^-1^[56].

Raman shift bands centered at 1315 [50, 57–60] and 1254 cm^-1^ [61] are attributed mainly due to nucleic acids, lipids, and collagen. CH_2_ and CH functional groups' vibration of lipids and protein respectively, attribute mainly to the band at 1450 cm^-1^ [52, 55]. The peak centered at 1365 cm^-1^ is attributed mainly to tryptophan [56] and guanine [62]. Cytosine contributes to the bands centered at 1284 cm^-1^ [62]. Amide III is represented at this peak, and also at the peaks centered at 1284 [61] and 1238 cm^-1^ [63]. RNA [13] and $\text{P}{\text{O}}_{2}^{-}$ [57] contribute to the peak centered at 1238 cm^-1^.

The 1185–1485 cm^-1^ wavenumber region is mainly attributed due to proteins [49, 64], lipids [65], and phosphate compounds [66, 67], via their **CH**_**2**_, **CH**_**3**_, and P = O functional groups, respectively.

Carbohydrates and polysaccharides absorption bands contribute mainly in the low wavenumber region below 1185 cm^-1^ [68], due to their functional groups C—O—C, C—O—P.

The major differences between the averages spectra belonging to the four biological systems investigated in this study occur in the high region of the spectrum in 1195–1730 cm^-1^ namely in the proteins ranges amide II and amide III shift bands. Generally, there is a significant reduction in the intensity of absorption at this region as a result of infection with any of the tested viruses.

**Fig 2:**
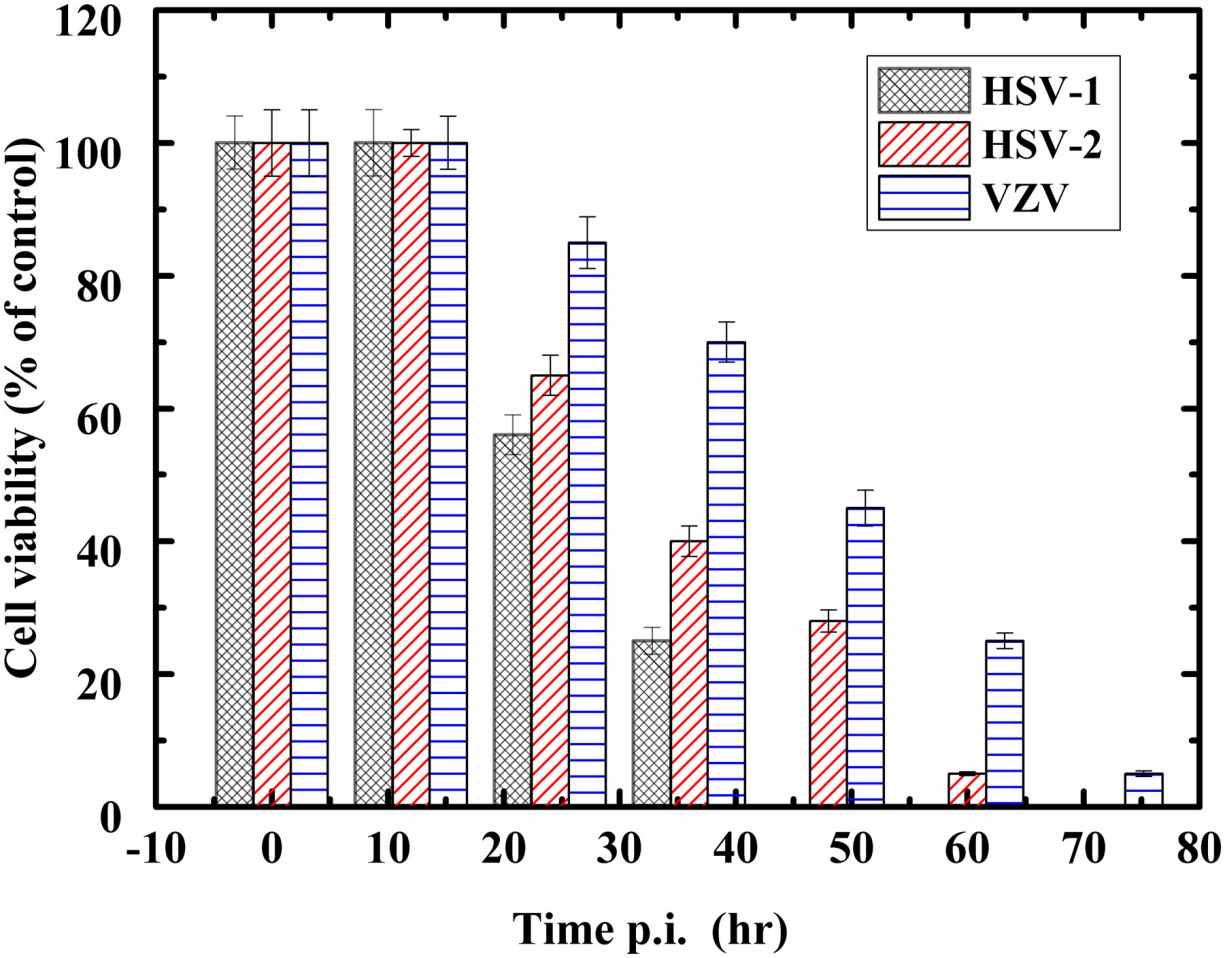
Effect of infection with herpes viruses on cell viability. The effect of infection with the different herpes viruses on cell viability. Vero cell monolayers were infected with 1 m.o.i. of either HSV-1, HSV-2, or VZV. Viability of the infected cells was evaluated at various times post infection (p.i.) by MTT test. Data are mean ± SD (n = 4).

Fig 2 describes Vero cells viability after infection with HSV-1, HSV-2, and VZV, separately. Vero cell monolayers were separately infected with a high titer (5 m.o.i.) of each one of the examined viruses and the viability of the infected cells was evaluated at different times post infection, using the MTT test [44]. The results are averages of four different experiments.

As can be seen from the figure, the HSV-1 virus infection manifests itself quicker than infections with the other viruses, with control cell survivability extinguished within 48 hours or less; control cell survivability after HSV-2 and VZV virus infections were reduced to less than 5% after 60 to 72 hours, respectively.

**Fig 3:**
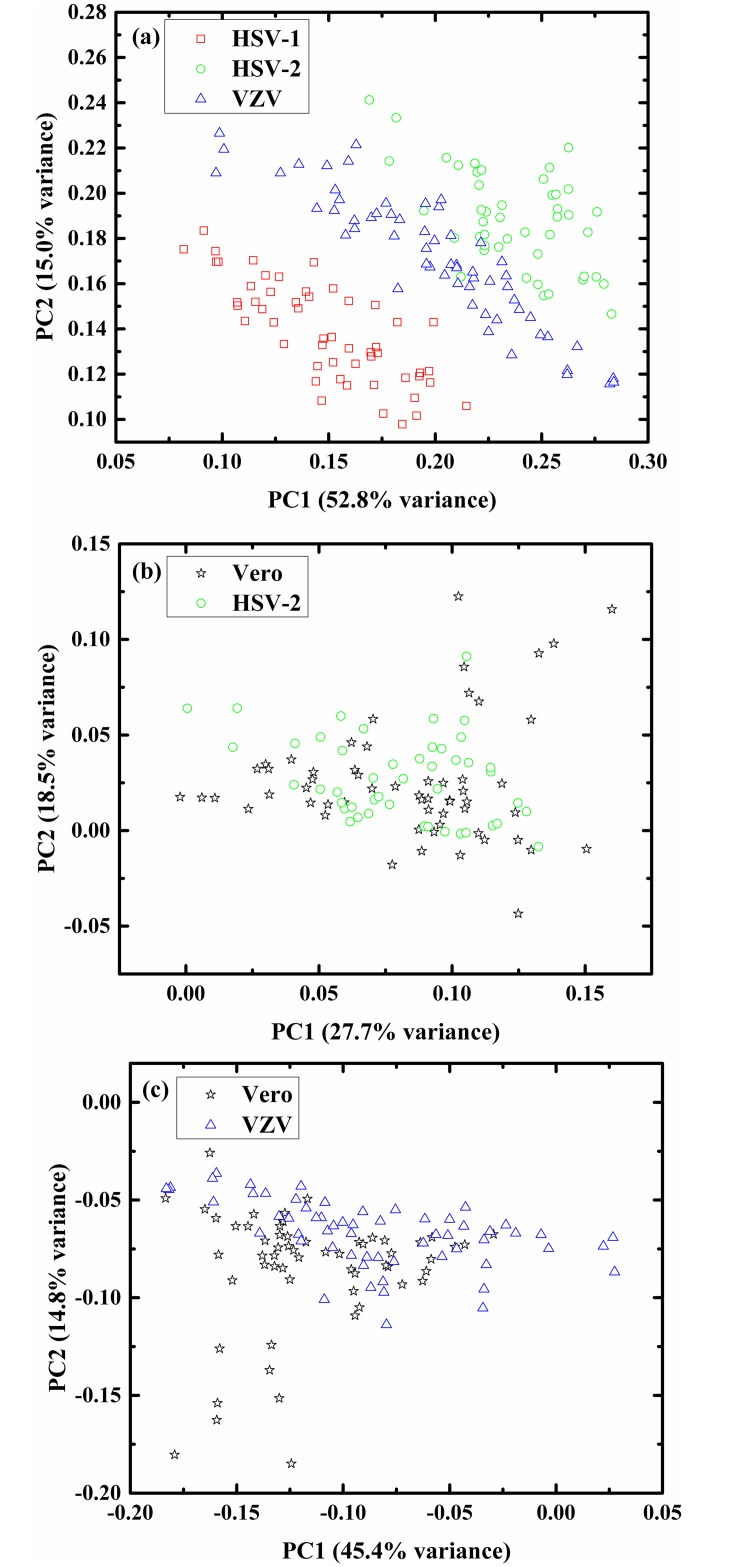
Plotting PC's scores calculated by PCA in two-dimensional plots. 2D figure of the different biological samples investigated in this study. In this figure the scores are shown for PC1 versus PC2: (a) HSV-1 infected Vero cells, HSV-2 infected Vero cells, and VZV infected Vero cells; (b) Vero cells, HSV-2 infected Vero cells; and (c) Vero cells, VZV infected Vero cells.

The spectra in Fig 1 are averages spectra. The individual spectra of the four different categories overlap, and that it is difficult to differentiate among them using simple methods like clustering and k-means; thus we used multivariate analysis. Using PCA, we reduced the dimension of the spectra from 688 to 9, making it easier to analyze them using different classifiers. As the purpose of PCA is dimensionality reduction, sometimes it is possible to classify the different categories using projection of the data at different planes, by generating 2D figures. For example, Fig 3 shows 2D plots for: (a) the three infected categories HSV-1, HSV-2 and VZV infected Vero cells, (b) two categories—control cells and HSV-2 infected cells, and (c) two categories—control cells and VZV infected cells. In Fig 3a the HSV-1 infected cells are totally separated from the HSV-2 and VZV infected cells categories, while there are some overlapped points between the HSV-2 and VZV infected cells categories. In Fig 3b, the data points of the two categories; control cells and HSV-2 infected cells are overlapped, and the same trend can be seen between the control cells and VZV infected cells (Fig 3c). Thus, we used the LDA classifier to classify the four biological systems on the data, after the PCA calculations.

**Fig 4:**
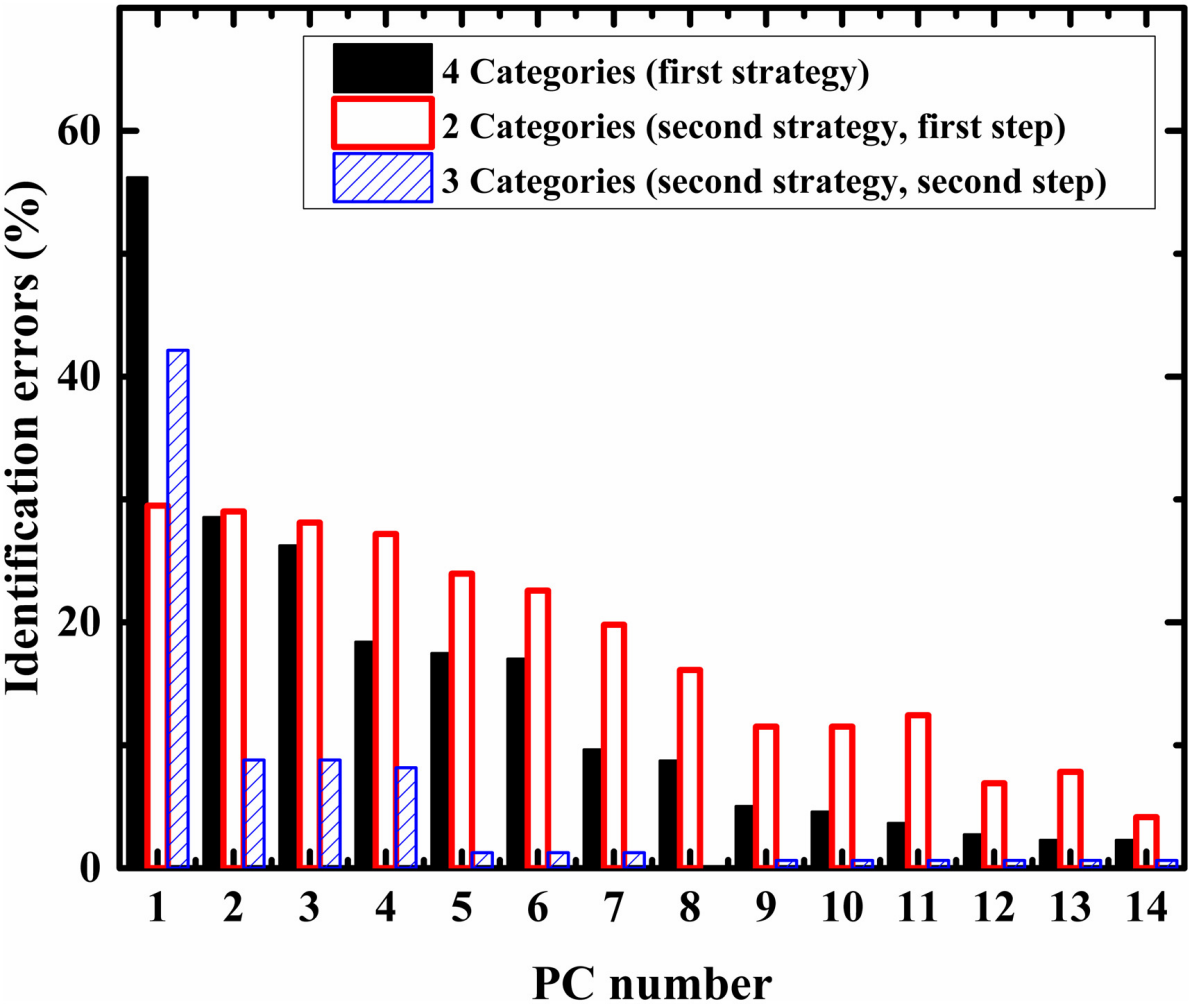
Performance of LDA calculation in different classification procedures. Identification errors, calculated by LDA using LOO algorithm, versus PC number, are presented in the figure. Black squares-the spectra were classified into the four categories of Vero, HSV-1, HSV-2, and VZV simultaneously. Red squares-the spectra were classified into two categories, control and infected. Blue square-the infected spectra were classified into three categories HSV-1, HSV-2 and VZV.

Fig 4 shows identification success in percentage, as a function of PC number. The identification success rates were estimated using the LOO algorithm. This algorithm is usually applied when the statistics are relatively small; here, the training set contained all but one of the measured spectra. The category of this left-out measurement is predicted using LDA and then compared to the known category. This procedure was repeated N times, but in each repetition a different measured spectrum was left out. The classifications were performed using two strategies. In the first strategy, the spectra were classified into four classes; Vero cells, HSV-1 infected Vero cells, HSV-2 infected Vero cells, and VZV infected Vero cells. In the second strategy, the spectra were classified into two steps; in the first step the spectra were classified into two groups, control (Vero cells) and infected cells (HSV-1, HSV-2 and VZV infected Vero cells). In the second step, the spectra of the infected group were classified into three classes: HSV-1 infected Vero cells, HSV-2 infected Vero cells, and VZV infected Vero cells.

As can be seen from Fig 4, for the LOO method to achieve more than 90% accuracy, a different PC number should be used, depending on the strategy chosen; for example using the first strategy (with 4 categories) 8 PCs are needed, while for the second stage step 2, two PCs are needed,

The identification success rates for the first and second strategies are listed in Tables 1 and 2 respectively.

**Table 1:** Performances of LDA calculations for the first strategy. The calculations were performed in order to differentiate between all four classes—control Vero cells, HSV-1 infected Vero cells, HSV-2 infected Vero cells and VZV infected Vero cells using 13 PCs.

	Vero cells	HSV-1 infected Vero cells	HSV-2 infected Vero cells	VZV infected Vero cells
Vero cells	55		3	
HSV-1 infected Vero cells		53		0
HSV-2 infected Vero cells	1		45	
VZV infected Vero cells	1			59

**Table 2:** Performances of LDA calculations for the second strategy, step 1: The calculations were performed in order to differentiate between two classes, control Vero cells and infected Vero cells. The infected Vero cells category includes HSV-1 infected Vero cells, HSV-2 infected Vero cells and VZV infected Vero cells, and uses 13 PCs.

	Vero cells	Infected Vero cells
Vero cells	51	7
Infected Vero cells	10	149

For the second strategy step 2, where the calculations were performed to differentiate among the three sub-classes of the infected category in the first stage of the second strategy, a 100% success rate was achieved using 9 PCs.

We tried to compare between all the different pairs of the four categories: control cells and HSV-1 infected cells; control cells and HSV-2 infected cells; control cells and VZV infected cells; HSV-1 infected cells and HSV-2 infected cells; HSV-1 infected cells and VZV infected cells; and HSV-2 infected cells and VZV infected cells.

The LDA calculations were performed for each pair, using the LOO approach for validation. The results of LDA calculations are presented in Table 3. The success rates of the differentiation among the different groups were calculated using different numbers of PCs.

**Table 3:** Identification success in percentage versus PC number, derived using LDA calculation for six pairs. The cumulative variance is displayed in parentheses.

Number of PCs	Vero cells-HSV-1 infected Vero cells	Vero cells-HSV-2 infected Vero cells	Vero cells-VZV infected Vero cells	HSV-1 infected Vero cells- HSV-2 infected Vero cells	HSV-1 infected Vero cells-VZV infected Vero cells	HSV-2 infected Vero cells-VZV infected Vero cells
PC1	91.9(48.8)	52.9(27.7)	61.0(45.4)	98(52.8)	67.3(48.9)	62.3(46.6)
PC1-PC2	98.2(63.1)	48.1(46.2)	71.2(60.2)	100(67.8)	99.1(67.4)	89.6(60.9)
PC1-PC3	100 (71.6)	65.4(57.3)	77.1(68.6)			88.7(67.9)
PC1-PC4		72.1(64.2)	83.1(72.9)			90.6(73.5)
PC1-PC5		77.9(73.2)	83.9(76.8)			88.7(77.9)
PC1-PC6		91.3(75.8)	91.5(80.1)			99.1(81.1)

**Fig 5:**
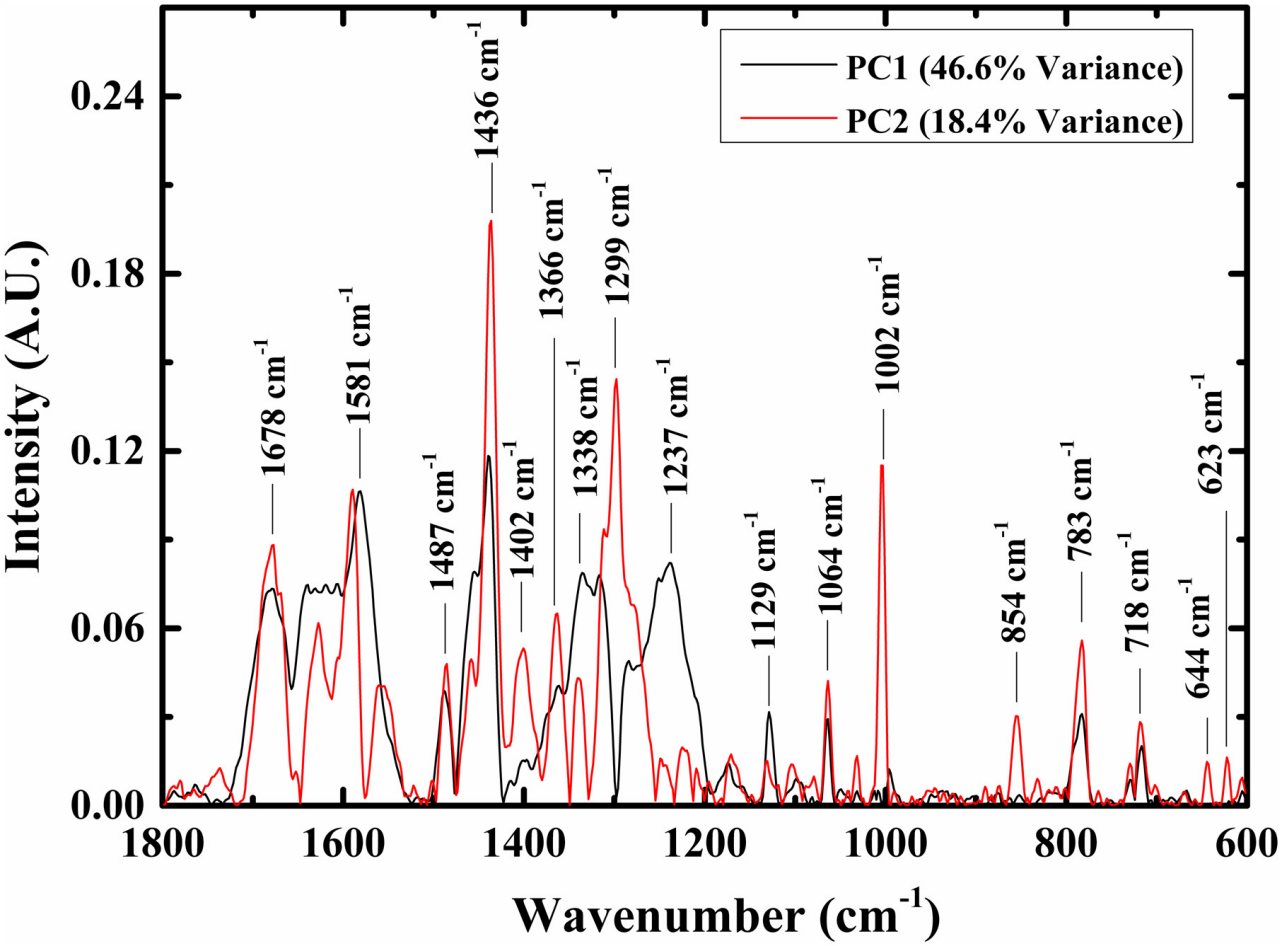
Loadings plots. Loadings 1 and 2 determined using PCA calculations (absolute values) are plotted in the 600–1800 cm^-1^ region. The major peaks and their centroids are displayed in the figure.

Analysis of the loadings may give some understanding of spectral features, which may contribute to the discriminant procedure [69, 70]. Using one PC it was possible to differentiate between the control Vero cells and the infected Vero cells with more than a 70% success rate. Thus, it may be suggested that the major bands in PC1 have major roles in the differentiation process (Fig 5). Absolute values of loadings 1 and 2 are shown in Fig 5; the dominant bands are labeled in the figure.

## Discussion

In our previous study [40], we successfully used Raman spectroscopy to classify HSV-1 infected Vero cells and normal uninfected. In this study, we examined the potential of Raman spectroscopy to identify and classify control uninfected Vero cells and cells infected with either of the three most common members of the herpes virus family (HSV-1, HSV-2, and VZV).

Enlarging the number of categories is a challenge for pattern recognition methods when the classes (tested samples) are very similar. For instance, HSV-1 shares very high similarities with HSV-2, both in their genome sequences (over 70% homology) and in their clinical symptoms [71, 72].

The Raman shifts spectrum is characteristic of the vibrational modes of the biological molecules—protein, nucleic acid, lipid, and carbohydrate molecules, due to their functional groups N—H, C = O, C-H, and P = O vibrations [50, 53, 54, 56–59, 61, 62, 73, 74]. As can be seen from Figs 1 and 5 (PC1), the major spectral differences between the infected Vero cells and the control cells occur in the 1195–1726 cm^-1^ region. Nevertheless, when LDA calculations were performed using the Raman spectra in this region, the performance of these calculations reflected in the classification success rates was slightly decreased when compared to the classification results using the 600–1726 cm^-1^ region. Thus, the 600–1195 cm^-1^ region is still important to the classification procedure. These observations are in accordance with the loading analysis shown in Fig 5. There are some Raman band shifts in the 600–1195 cm^-1^ region (much smaller than the Raman shifts in the high region); these still could improve the classification performance of the LDA calculation. These band shifts are centered at 1129 cm^-1^, 1064 cm^-1^, 783 cm^-1^, and 718 cm^-1^, and contributed mainly due to carbohydrates and polysaccharides absorption bands [68], due to their functional groups C—O—C, C—O—P.

As can be seen from Fig 1, the spectral intensities of all infected cells with either of the tested viruses are lower in the 1195–1380 cm^-1^ and 1537–1637 cm^-1^ ranges compared to the control uninfected cells, while they have slightly higher intensities in the 1380–1500 cm^-1^ rang.

The herpes viruses are known as lytic viruses, which cause the termination of all metabolic activities of the host cells after infection, by blocking synthesis of cellular proteins and causing cellular DNA degradation [75, 76]. In fact, these viruses use all cellular stores for their advantage, thereby synthetizing all viral components that are required for their own replication. Thus, infection with these herpes viruses may reduce the amounts of cellular contents such as protein, lipid, and nucleic acid molecules, as reflected in the spectral intensities in Raman shift spectra.

Using PCA the dimensions are reduced to a few PCs (loadings). Each spectrum is represented as a superposition of three loadings (PCs) in the new domain. For example, using five PCs, each spectrum was identified by five numbers named as the coefficients of the loadings. The purpose of PCA calculation is dimensionality reduction, and the projection of the transformed data at certain planes often yields good separation (2D figures) (Fig 3a). For the classification procedure we used the LDA method. The LDA calculation was designed applying the LOO method, which is a common method of cross-validation that has been extensively explored in machine learning and is primarily used to estimate the error in a small sized populations [77, 78]. The classification procedure was performed using two strategies. In the first strategy, the classification was performed among the four biological systems investigated in this study simultaneously. Using 13 PCs it was possible to achieve a classification success rate in excess of 96%. Keeping in mind that the infections with these herpes viruses are biologically similar, these results are considered very good [40, 79–82]. In the second strategy, the classification procedure was performed in two stages. In the first stage the results were classified into two categories, control Vero cells and infected Vero cells. The infected cells include the HSV-1, HSV-2, and VZV infected cells. In this first stage of the second strategy, using 13 PCs made it possible to achieve a classification success rate in excess of 93%.

Applying PC1, the identification errors using two classes of classification, control-infected (second strategy, first stage), were significantly lower than the errors in the four categories classification (first strategy) (Fig 3). This was due to the differences in the number of classes. However, when using more PCs, the trend was changed, which is not surprising, because the intra-variance among the infected group is higher than the intra-variance of each of the four groups. This is because the infected group includes HSV-1, HSV-2, and VZV infected Vero cells.

In the second stage of the second strategy, the infected group was simultaneously distributed into three classes HSV-1infected cells, HSV-2 infected cells, and VZV infected cells. Using two PCs, it was possible to achieve a classification success rate in excess of 98%.

We tried to compare among the six possible couples of the four biological samples: control cells and HSV-1 infected cells; control cells and HSV-2 infected cells; control cells and VZV infected cells; HSV-1 infected cells and HSV-2 infected cells; HSV-1 infected cells and VZV infected cells; and HSV-2 infected cells and VZV infected cells.

By the nature of PCA, all the data is transformed into a new basis called loadings (PCs), which suggest better classification between the classes. The variance among the classes is captured by the different loadings (PCs). In the new space, each spectrum is calculated as a linear combination of the new basis (loadings). The loadings are arranged in descending order according to their variance; thus, PC1 has the largest variance, followed by PC2, and so on.

As can be seen from Table 3, the largest spectral differences occur between HSV-1 infected cells and HSV-2 infected cells, because when using the first PC1, a 98.0% success rate was achieved. The spectral differences between HSV-1 infected cells and VZV infected cells are also large, because a 99.0% success rate was achieved by using the first two PCs. This result is somewhat surprising because, as mentioned above, HSV-1 and HSV-2 are relatively very close to each other both in their genomes and in their clinical symptoms. More study is required in order to understand the reason for this spectral variance between these viruses. Of course, differences occurred in all different groups because we can differentiate between all the couples and the spectral differences are relative issues

This study proved the potential of Raman spectroscopy as a promising method for successful identification of infected cell cultures with either of the tested herpes viruses. As mentioned in the introduction section, it is highly important to identify the cause of such infections in order to proscribe effective treatment. There are distinct advantages of the Raman spectroscopic method in tandem with multivariate analysis over the classical methods-it takes a short time (few minutes) to determine the cause of the infection and it is highly objective and computerized.

When classification was among the three infected categories, the classification rate was 57.9% using PC1 only, and increased to 91.2% using PC1 and PC2. Thus, we hypothesize that the bands that appear in PC2 but do not appear in PC1 have special importance to the classification procedure among the three infected categories and are listed in Fig 5. The major peaks are centered at 1436 cm^-1^ and contributed mainly to proteins and lipids due to their CH vibrations [52, 55]. Amide III and cytosine are represented at this peak and contributed also to the bands centered at 1299 cm^-1^ [62], while the bands centered at 1002 cm^-1^ are due to phenylalanine in proteins. The bands centered at 854 cm^-1^ are due to DNA [69].

## Conclusions

It was possible to differentiate among the infections caused by three different herpes virus types (HSV-1, HSV-2 and VZV) with a high rate of success. This was accomplished through Raman spectroscopy that was analyzed using multivariate analysis. The method is objective, computerized and fast.

## Supporting Information

S1 Data An excel file, "S1 data.xls" was uploaded. This file contains the raw spectral data after spectral manipulations. The file contains four sheets: "Vero" sheet contains the data for the control Vero cells; "HSV1" sheet contains the data for the Vero cells infected with HSV1 virus; "HSV2" sheet contains the data for the Vero cells infected with HSV2 virus and "VZV" sheet contains the data for the Vero cells infected with VZV virus.
(XLS)
